# Hypoalbuminemia differently affects the serum bone turnover markers in hemodialysis patients

**DOI:** 10.7150/ijms.39158

**Published:** 2019-10-21

**Authors:** Cai Mei Zheng, Chia Chao Wu, Chien Lin Lu, Yi Chou Hou, Mai Szu Wu, Yung Ho Hsu, Remy Chen, Tian Jong Chang, Jia Fwu Shyu, Yuh Feng Lin, Kuo Cheng Lu

**Affiliations:** 1 Graduate Institute of Clinical Medicine, College of Medicine, Taipei Medical University, Taipei 11031, Taiwan; 2 Division of Nephrology, Department of Internal Medicine, School of Medicine, College of Medicine, Taipei Medical University, Taipei 11103, Taiwan; 3 Division of Nephrology, Department of Internal Medicine, Shuang Ho Hospital, Taipei Medical University, New Taipei City 235, Taiwan; 4 Division of Nephrology, Department of Medicine, Tri-Service General Hospital, National Defense Medical Center, Taipei 114, Taiwan; 5 Division of Nephrology, Department of Medicine, Fu Jen Catholic University Hospital, School of Medicine, Fu Jen Catholic University, New Taipei City 242, Taiwan; 6 Division of Nephrology, Department of Medicine, Cardinal-Tien Hospital, School of Medicine, Fu Jen Catholic University, New Taipei City 23155, Taiwan; 7 Chief, Kidney Dialysis Center, Hasuda Hospital, Negane, Hasuda City, Saitama, 3490131, Japan; 8 Graduate Institute of Life Sciences, National Defense Medical Center, Taipei 114, Taiwan; 9 Performance Appraisal Section, Secretary Office, Shuang Ho Hospital, Taipei Medical University, Taipei, Taiwan; 10 Department of Biology and Anatomy, National Defense Medical Center, Taipei 114, Taiwan

**Keywords:** renal osteodystrophy, hypoalbuminemia, bone turnover markers, inflammation, hemodialysis patients

## Abstract

Renal osteodystrophy (ROD) represents bone disorders related to chronic kidney disease (CKD) and several bone biomarkers are used clinically to predict ROD in CKD and hemodialysis (HD) patients. Serum albumin associates with inflammation other than nutritional status in these patients. Chronic inflammation is proved to relate with bone loss, however, the influence of hypoalbuminemia on bone biomarkers is still unclear. In this study, we evaluated the pattern of bone biomarker changes and further studied the influence of hypoalbuminemia on these biomarkers. A total of 300 maintenance HD patients were evaluated and 223 HD patients were included in the study. The patients were grouped according to serum parathyroid hormone (PTH) levels (PTH ≤150 pg/mL, PTH 150-300 pg/mL, PTH 300-600 pg/mL and PTH >600 pg/mL). Bone biomarkers and inflammatory markers were measured and their relation with PTH levels was determined. Significantly increased interleukin-6 (IL-6) and lower albumin levels were noted among PTH>600 pg/mL group. Bone turnover markers were significantly higher in PTH >600 pg/mL group (p< 0.05). Hypoalbuminemia significantly increased the fibroblast growth factor-23 (FGF-23) and procollagen type 1N-terminal propeptide (P1NP) in PTH ≤150 pg/mL, PTH 150-300 pg/mL, PTH 300-600 pg/mL groups, whereas no such relation was noted among PTH> 600 ng/dL group. In conclusion, hypoalbuminemia represents a chronic inflammation which differently relates to bone turnover markers according to serum PTH levels in SHPT patients. Thus, serum albumin measurement should be considered in determining bone disorders among these patients.

## Introduction

Chronic kidney disease-mineral bone disorder (CKD-MBD) related renal bone disorder, also known as renal osteodystrophy (ROD), is a clinically unique bone disease of CKD and HD patients and distinct from normal aging-related osteoporosis in the general population. ROD relates with abnormalities of bone turnover, mineralization, volume, linear growth, and strength and results in both bone quantity and bone quality loss 1. Bone biopsy is the gold standard for definitive diagnosis of ROD 2. Due to its invasive nature, cost and overall complexity, it is rarely done nowadays in clinical practice. Clinically, the fracture risk assessment is done by the quantitative analysis of bone mineral density (BMD) by dual-energy X-ray absorptiometry (DXA). However, DXA only assesses the bone quantity and does not give the information about bone quality. The micro-architectural quality loss including micro-damage and remodeling rates are needed to be identified. Two-dimensional DXA discriminates cortical and cancellous bone by determining the bone geometry. Quantitative computed tomography (QCT) is recently known for more accurate assessment of bone quality 3, 4. However, these imaging techniques have limited accessibility due to high cost and bulky device. Several bone related biomarkers have been used to determine the bone turnover status in CKD and HD patients. However, these bone turnover markers still lack evidence to represent the bone status in CKD-MBD, which limits these patients from necessary treatments.

Serum intact PTH (iPTH, PTH) has long been regarded as the principal biomarker for assessing the bone turnover in diagnosis and monitoring of ROD 5, 6. Although PTH is a key player in pathogenesis for SHPT, the measurement of PTH is more reflective of parathyroid activity rather than of the bone status in CKD-MBD 7. Some studies proved the relation between serum PTH and bone histomorphometric parameters especially the bone formation rate 7-10. The nature of bone disorders in CKD changes from the predominant “high turnover”/high PTH osteitis fibrosa lesions in the 1960s to 1980s to adynamic bone lesions in 1990 to 2010s due to increased prevalence of aging, diabetes mellitus and increased use of calcium loading phosphate binders 11. Such a paradigm shifts together with intrinsic low specificity, little relation was noted between serum PTH and bone formation rates except in those with extremes of PTH values (e.g., >600 ng/L and <100 ng/L)^12^. Thus, measuring the serum PTH alone has a higher sensitivity but a lower specificity in assessment of high bone turnover disorders 12, 13. Many bone turnover markers are under consideration alone or with PTH to determine the turnover status more accurately without renal biopsy, which is painful, invasive and not feasible in clinical grounds.

However, most bone turnover markers are still not widely used due to lack of definitive sensitivity and/or specificity. Total alkaline phosphatase (ALP), bone-specific alkaline phosphatase (BALP),osteocalcin, and procollagen type 1N-terminal propeptide (P1NP) act as bone formation markers and measure osteoblast function 14, 15. Bone resorption markers such as tartrate-resistant acid phosphatase 5b (TRAP-5b) and C-terminal telopeptides of type I collagen (CTX) represent osteoclast number and function 15. However, CTX measurement is not recommended in CKD patients due to its abnormal accumulation related with impaired renal function 16. Serum TRAP-5b concentration is not influenced by kidney function and is regarded as the best bone resorption marker among uremic patients 17, 18. The receptor activator of nuclear factor NF-κB ligand (RANKL) and its membrane-bound receptor (RANK) in the osteoclast precursor cells 19 determine the osteoclast activation, differentiation and survival 20-22. Osteoprotegerin (OPG) inhibits bone resorption by binding to RANKL 23, 24 and the balance between levels of OPG and RANKL regulates osteoclastic activity 25. A disturbance in wingless (wnt) signaling is also noted among CKD patients. Wingless (wnt) signaling regulates the osteoblast differentiation during bone remodeling. Increased osteocytic sclerostin expression and repressed osteocytic wnt/β-catenin signaling associates with low turnover bone status since early CKD 26. With progressively higher PTH levels, PTH overcomes the peripheral PTH resistance and wnt inhibitors. PTH suppresses skeletal sclerostin expression and generates a high bone turnover state 26. PTH also mediates the interaction between wnt signaling and its inhibitors sclerostin and Dkk-1. PTH/PTH1R complex binds and phosphorylates the wnt co-receptor LDL-receptor-related protein (LRP-6) and stabilizes β-catenin without any wnt binding 7. Activation of the PTH receptor down-regulates the sclerostin and Dkk-1 and activates intracellular wnt signal transduction 8-11. Earlier works 12, 13 revealed that plasma sclerostin levels positively correlated with serum phosphate, FGF23 levels and negatively correlated with PTH levels in hemodialysis patients. Wnt3a and wnt10b, acting through canonical signaling; and wnt16, acting through both canonical and noncanonical signaling, to induce production of OPG in osteoblasts and further inhibits osteoclast differentiation.

Clinically, chronic inflammation plays an important role in development of osteopenia and osteoporosis. Various studies report an increased risk of osteoporosis and bone loss in various inflammatory conditions including gout, osteomyelitis, rheumatoid arthritis, ankylosing spondylitis, etc 27-31. Inflammatory cytokines such as tumor necrosis factor (TNF)-α and interleukin (IL)-6 are elevated as a result of increased bone resorption 32, 33. On the other hand, systemic inflammation and serum albumin concentration significantly relate with mortality among CKD and HD patients 34, 35. HD patients experience the dialysis process associated inflammation 36, 37 in addition to those related with underlying disease conditions. Chronic inflammatory markers such as CRP, IL-6, and TNF-α were elevated in these patients 38. Clinically, chronic IL-6 elevation is more predictive of inflammation among HD and pre-dialysis CKD populations 39 compared to CRP and TNF-α levels 39. Hypoalbuminemia is also highly prevalent among HD patients (25-50%) and also associated with high morbidity and mortality 34, and a greater risk of mortality noted particularly when serum albumin <3.8 g/dL 40-42. Many studies reveal that hypoalbuminemia represents a marker of systemic inflammatory response rather than poor nutrition among dialysis patients 34, 43, 44. Many comorbid conditions commonly associate with hypoalbuminemia in dialysis patients 45 include diabetes mellitus 46, peripheral vascular disease, smoking, neoplasms, etc. Chronic inflammation and susceptibility to infection relates with increasing resting energy expenditure (REE) and results in body tissue mass wasting and hypoalbuminemia in dialysis patients 47, 48. Metabolic acidosis suppress albumin synthesis 49 and increase skeletal muscle catabolism 50, 51 in these patients. Analysis of a national dataset find a significantly increased risk of osteoporosis among general population with hypoalbuminemia; with albumin <3.5g/dL patients has 5.37 fold at femoral neck, 12.46 fold at total femur, and 4.59 fold at lumbar spine higher risk of osteoporosis as compared to those with albumin >4 mg/dL 52.

However, how the hypoalbuminemia influences the levels of bone turnover markers in HD patients is still unknown. We studied the HD patients and grouped them according to serum PTH levels and different bone turnover biomarkers were measured. Then, we studied the relation between serum albumin and bone turnover markers. Finally, we hope to predict more accurately about the bone turnover status using these biomarkers in HD patients.

## 2. Results

### 2.1. Baseline Patient Characteristics

Table 1 shows the demographic characteristics of the study patients. All the patients were under HD for more than 3 months. Age, sex, dialysis duration and body mass index (BMI) were not significantly different. The patients were sub-grouped according to PTH levels (PTH ≤150 pg/mL, PTH 150-300 pg/mL, PTH 300-600 pg/mL and PTH >600 pg/mL), respectively. Serum albumin level was significantly decreased, whereas inflammatory mediator IL6 was significantly increased among PTH>600 pg/mL group than other PTH groups. Serum phosphate levels and FGF 23 were significantly increased among those with PTH 300-600 pg/mL and PTH >600 pg/mL groups. No significant differences were noted regarding the dialysis adequacy Kt/V and protein intake among different groups. Serum hematocrit, hemoglobin, calcium levels were also not significantly differ.

### 2.2. Changes in Bone turnover Markers among Different PTH Groups

The bone turnover markers were determined according to PTH levels among these patients (Table 1). Serum Alk-P, OPG, wnt 10b, P1NP and TRAP-5b levels were significantly higher in PTH >600 pg/mL group (p< 0.05) compared to other groups. Other bone markers including wnt 16, sclerostin and DKK1 levels did not differ significantly among various PTH groups (Table 1).

### 2.3. Relation between Serum Albumin and Inflammatory Markers in Different PTH Groups

A significant negative correlation was noted between IL-6 and serum albumin levels among PTH ≤150 pg/mL, PTH 300-600 pg/mL and PTH >600 pg/mL groups and no relation or a non-significant positive relation was noted in PTH 150-300 pg/mL group(Table 2).

### 2.4. Relation between Serum Albumin levels and Bone Turnover Markers in Different PTH Groups

Relation between serum albumin levels and bone turnover markers was also evaluated among different PTH groups (Table 2). Bone formation marker total alkaline phosphatase (ALP) was negatively correlated with albumin levels in different PTH groups, however, only significant in PTH 150-300 group. Procollagen type 1 N-terminal propeptide (P1NP) level had a significant negative relation with albumin in PTH<150 group and no significant relation was noted in other groups. No significant relation was noted between bone resorption marker tartrate-resistant acid phosphatase 5b (TRAP-5b) with albumin levels. Osteoclast regulator OPG had a significant positive relation with albumin levels in all PTH groups except PTH>600 group. Similarly, Wnt signaling mediator such as wnt 10b was negatively correlated with albumin in PTH≤ 600 pg/mL groups though significant only in those with PTH <150 and 300-600 groups. Plasma sclerostin levels positively correlated with serum albumin levels in those with PTH>600 pg/mL group alone (Table 2).

We determined the influence of hypoalbuminemia (albumin≤3.5 g/dL) and normoalbuminemia (albumin>3.5 g/dL) on the bone formation and resorption markers in different PTH groups (Table 3). Serum FGF23 levels were significantly increased in hypoalbuminemia PTH 150-300 and 300-600 groups than normoalbuminemia patients. Although not significant, an increased in FGF23 levels were also noted in PTH≤150 pg/mL group. However, no such relation was noted between albumin and FGF23 levels and even became reverse in those with PTH>600 group. Similarly, P1NP levels were found to be significantly increased among those with PTH ≤150 pg/mL, 150-300, 300-600 groups, whereas no significant increase was noted and even lower among those with PTH >600 pg/mL groups. No significant relation was noted between hypoalbuminemia and other bone turnover markers.

## 3. Discussion

As described, bone turnover disorders are major causes of bone quantity and quality loss among dialysis patients 1. Serum albumin is a marker of systemic inflammatory status among dialysis patients; and it is well known that hypoalbuminemia independently associated with bone loss among general population 53, 54. To our knowledge, there is no previous study regarding the influence of hypoalbuminemia on these bone turnover markers among dialysis patients. The present study demonstrated that significant hypoalbuminemia and inflammation occurred among PTH>600 pg/mL group compared to other PTH groups. Bone turnover markers including Alk-P, Wnt 10b, P1NP and TRAP-5b levels were significantly higher in PTH >600 pg/mL group (p< 0.05) as compared to other groups (Table 1). Interestingly, a differential correlation was noted between serum albumin and P1NP levels with a negative relation in PTH≤150 pg/mL, 150-300, 300-600 pg/mL groups, and was reversed in those with PTH>600 pg/mL group (Tables 2 &3). Similar relation was noted between serum albumin and FGF 23 levels (Tables 2 &3). Although PTH>600 pg/mL group had significantly higher FGF23 and P1NP, their levels not relate with serum albumin levels. These findings proved that in patients with PTH≤150 pg/mL, 150-300, 300-600 pg/mL groups, hypoalbuminemia might influence the measurement of FGF23 and P1NP levels. In severe hyperparathyroidism (SHPT) (PTH>600 pg/mL) patients, these levels were elevated regardless of serum albumin levels.

We found that the serum albumin level was significantly decreased and inflammatory mediator IL6 was significantly increased among PTH>600 pg/mL group than other PTH groups. No significant differences were noted regarding the dialysis adequacy Kt/V and protein intake among different groups (Table 1). Thus, protein malnutrition alone can't explain the cause of hypoalbuminemia in dialysis patients. Previous studies revealed that hypoalbuminemia prevalent in ESRD patients resulted from multiple processes including protein energy wasting (PEW) , inflammation and/or plasma volume expansion. Albumin serves as a negative acute-phase reactive protein 55, and its concentration is reduced during inflammation even in the absence of malnutrition 56, 57. A significant negative association was noted between serum albumin and IL-6 levels in PTH≤150 pg/mL, 300-600 pg/mL and >600 pg/mL groups, whereas no relation or non-significant positive relation in 150-300 pg/mL group (Table 2). Whether other conditions including malnutrition and uremic toxins interplay together in this relation is still unclear. Similar association was also found in a previous study on hemodialysis patients 58 and we deduce that inflammation plays an important role in hypoalbuminemia among our patients.

In consistent with previous studies 59, 60, significantly higher levels of FGF 23 and phosphate are noted in our cohort especially among those with higher PTH levels (Table 1). Increased FGF 23 level in CKD patients is related with stimulation of FGF23 gene expression by PTH 61, loss of FGF 23 transcriptional inhibitors 62 and chronic phosphate load 63. Normally, FGF23 decrease PTH gene expression and parathyroid cell proliferation 64, 65, however, hyperplastic parathyroid glands in uremia patients resist to FGF23 inhibitory action due to low expression of both klotho and FGF 23-receptor complex 66-68. A persistently elevated FGF-23 is also responsible for deterioration of bone mineralization 69-71. Higher PTH groups also revealed higher mean levels of bone formation markers P1NP, Alk-P and bone resorption marker TRAP-5b as a result of increased bone turnover. Progressively higher PTH levels upregulate RANKL m-RNA and inhibit OPG gene expression in bone marrow stromal osteoblasts 72. Previous studies also noted a significantly increased serum OPG level among predialysis and dialysis patients 73. An OPG increment in uremic patients might protect against intensive bone loss by inhibiting osteoclastic activity and reducing the RANKL level 74. We found that the OPG levels significantly lower and RANKL/OPG complex non-significantly higher among PTH>600 group, which explained the resistance for PTH and decreased protection from OPG among those with PTH >600 group. PTH also mediates the interaction between Wnt signaling and its inhibitors sclerostin and Dickkopf-1 (Dkk-1). PTH/PTH1R complex binds and phosphorylates the Wnt co-receptor LDL-receptor-related protein (LRP-6) and stabilizes β-catenin without any Wnt binding 75. Activation of the PTH receptor down-regulates sclerostin and Dkk-1 and activates intracellular wnt signal transduction 76-79. Serum sclerostin and DKK1 levels were reduced with increasing PTH levels. Interestingly, we found that Wnt10b levels are proportionately increased with PTH levels. This suggests that a possible compensatory Wnt 10b release in response to PTH occurs among these patients despite previous studies 80, 81 revealed that only intermittent PTH increases Wnt 10b expression. Though metabolomics studies revealed that Wnt 10b was reduced in CKD patients 74, herein, we proved that it was associated with PTH levels. Another bone anabolic Wnt 16 level was reduced among all patients regardless of serum PTH levels. A previous study revealed osteoblast-derived Wnt 16 increased OPG and inhibits osteoclastogenesis 82. This was not occurred among our hemodialysis patients since we found discordance between Wnt 16 and OPG levels.

Later, we found that serum FGF 23 and P1NP levels were differentially affected by serum albumin levels in various PTH groups (Tables 2 & 3). In patients with ≤150, PTH 150-300, PTH 300-600 groups, a significantly higher FGF23 was noted among hypoalbuminemic patients compared to those with normal albumin levels. This finding might be due to the influence of hypoalbuminemia in disturbance of bone deterioration by increasing FGF23. This was not found in PTH>600 groups which might explain the bone effects related with severely high PTH overcome the hypoalbuminemia. A significant lower OPG level was noted among hypoalbuminemic patients compared with normoalbuminemia in PTH 150-300 and 300-600 groups (Table 3). This explained that the protective actions of OPG on osteoclast inhibition were reduced and osteoclast activity was increased in hypoalbuminemia. It was also evident by increased pro-osteoclastic inflammatory marker IL-6 among our patients. Hypoalbuminemia, a mediator of chronic inflammation, directly activates the osteoclasts and suppress osteogenesis by activating NF-κB signaling pathway 74, 83, 84 together with other inflammatory cytokines. An increase in the RANKL/OPG ratio represents an imbalance between osteoclastic and osteoblastic activity and responsible for increased bone resorption 85. Characteristically, these patients revealed a significantly higher bone formation marker P1NP than normoalbuminemic ones in all PTH groups except PTH>600 group. Since P1NP representative of osteoblast activity, and it was deduced that higher osteoblastic activities related with hypoalbuminemia among these PTH groups (Table 3). These findings might be explained by the occurrence of osteoblast activation among hypoalbuminemia and related chronic inflammation. This is consistent with a previous study 86, which also proved that elevated inflammatory IL-6 related with osteoblast activation independently and in opposition to PTH actions. With severely high PTH levels (PTH>600), the influence of hypoalbuminemia on P1NP was no longer detected and overwhelmed by influence of PTH and uremic toxins on bone cells.

The study had several limitations. We did not evaluate bone biopsy in our study due to technical feasibility which became major limitation. The results were not applicable to whole HD population due to small sample size.All our patients were Taiwanese HD patients, which also difficult to generalize in other population. However, we found the significant impact of serum albumin levels on hyperparathyroidism bone metabolism. Patients were grouped according to serum PTH levels, which had high sensitivity and low specificity. We measured intact PTH levels (iPTH) which is recently proved to be related more with oxidative stress other than biological PTH activity instead of measuring non-oxidized PTH 7, 87. However, a discrepancy was noted in our patients with severe PTH>600 group compared to other PTH groups, and this might be explained by a different mechanism other than serum PTH levels. Bone mineral density was also not measured in study; however, our aim was to evaluate the relation between serum albumin and bone markers among these patients, which also did not correctly determine by bone mineral density. The study was a cross-sectional study and not evaluates the progressive changes in bone turnover markers among individual patients. Future studies are required to validate the relation of hypoalbuminemia, inflammation and bone turnover markers among CKD and dialysis patients.

## 4. Materials and Methods

### 4.1. Study Patients

A total of 300 patients undergoing maintenance hemodialysis in Cardinal Tien Hospital, Fu-Jen Catholic University and Shuang-Ho Hospital-Taipei Medical University Hemodialysis Unit were included in the study. 23 patients were excluded because they were within 6 months of starting hemodialysis. A further 18 patients treated with parathyroidectomy or calcimimetic agent cinacalcet therapy were excluded. 22 patients had a limited life expectancy due to one, or a combination of terminal malignancy at baseline (n=7), end-stage liver disease (n=4), end-stage cardiac disease (n=8), active infection (n=3) and 14 patients with missing information about starting dates of dialysis were also excluded. A total of 223 patients met our inclusion criteria and included in the study after informed consent.

**Figure 1:**
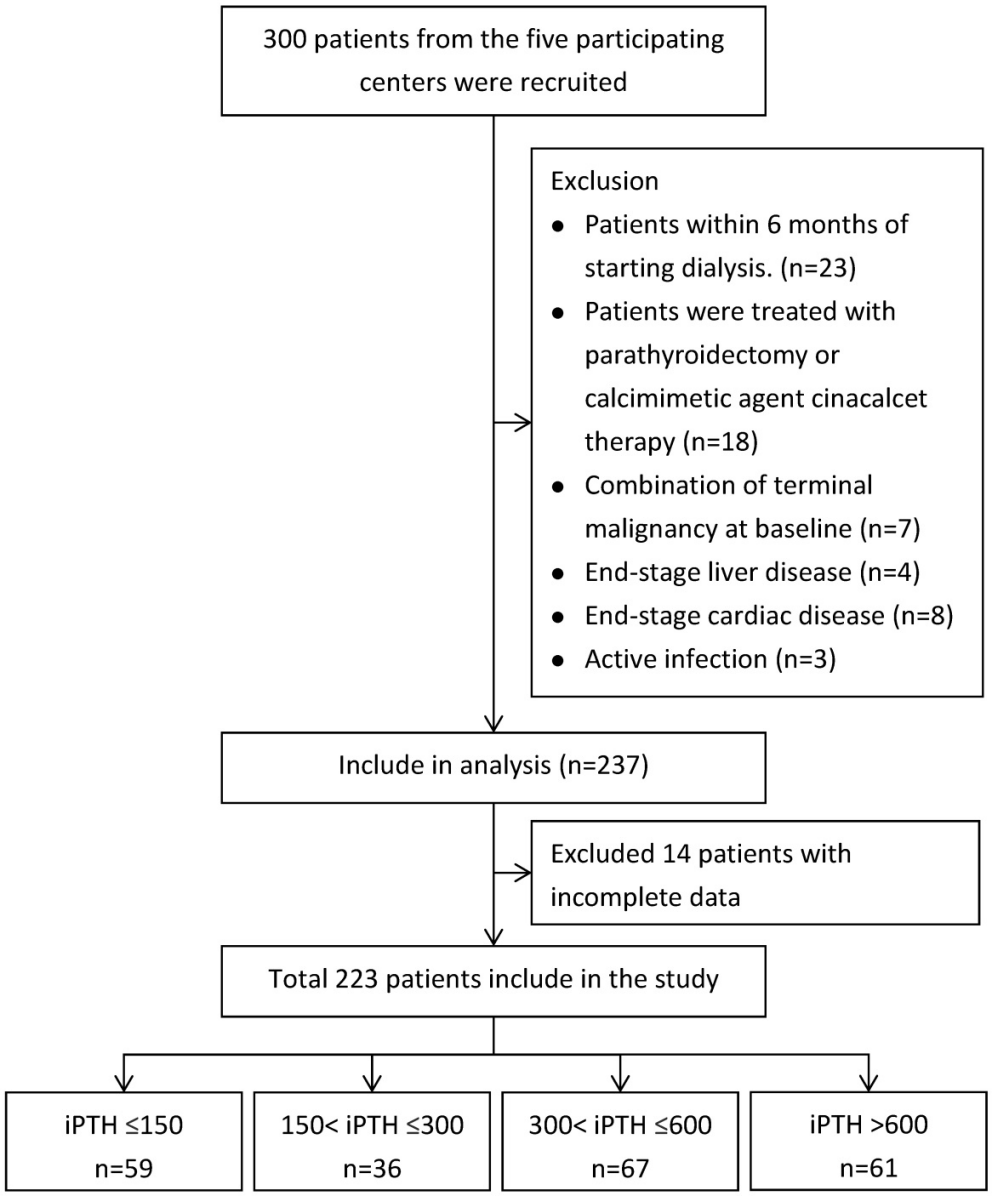
Flowchart of Patients Selection.

The patients were grouped according to PTH levels (PTH≦150, PTH 150-300, PTH 300-600 and PTH>600) (Figure 1). Baseline data and clinical parameters were listed in Table 1. Bone biomarkers including total alkaline phosphatase (Alk-P), Fibroblast growth factor 23 (FGF 23), procollagen type 1 amino-terminal propeptide (P1NP), tartrate-resistant acid phosphatase 5b (TRAP 5b), wingless 10b (wnt 10b), wnt 16, osteoprotegerin (OPG), RANK-L, beta-catenin, DKK-1, sclerostin (SOST) and routine biochemical parameters albumin, hemoglobin, Calcium (Ca), Phosphate (P) and total alkaline phosphatase (Alk-P) were measured and compared the changes according to PTH levels among four groups (Tables 1 & 2). Clinical data including values for urea Kt/V were determined. Dietary protein intake was also determined by calculating protein catabolic rate (PCR) by urea kinetics modeling 88. Single-pool model urea kinetics was used to estimate the normalized PCR (nPCR). This study was approved by the Human Ethical Committees of Cardinal Tien Hospital, Fu-Jen Catholic University and Shuang Ho Hospital, Taipei Medical University. Written informed consent was obtained from all participants.

### 4.2. Sample collection and biochemical parameters

Clinical characteristics of the patients, including age, gender and dialysis duration were obtained from medical records. Blood samples were collected into EDTA containing tubes and were processed within 1 h after venipuncture. Samples were subsequently centrifuged for 7 min at 1600 g. Concentrations of plasma calcium, phosphate, albumin, alkaline phosphatase (Alk-P) and other biochemistry parameters were measured by standard laboratory techniques with an automatic chemistry analyser (Synchron LXi-725; Beckman Coulter Inc., Brea, CA, USA). White blood cells, haemoglobin, and haematocrit were measured by automated hematology analysers XN 9000 (SYSMEX, KOBE, Japan).

### 4.3. Cytokines enzyme-linked immunosorbent assay

Plasma cytokine IL-6 was determined Human Instant Enzyme-Linked Immunosorbent Assay (ELISA) kit (eBioscience, San Diego, CA, USA) according to manufacturer's instructions. Serum was also separated to test the bone biomarkers such as wnt 10b, wnt 16, sclerostin (SOST), Dickkopf-related protein 1(DKK-1), Tartrate-resistant acid phosphatase 5b ( TRAP5b), Procollagen 1 N-terminal Propeptide (P1NP) and Fibroblast growth factor 23 (FGF-23) using commercially available ELISAs. (USCN: wnt 10b, P1NP; Cusabio: wnt 16; sclerostin, DKK-1, FGF-23; Quiedl: Trap5b; Biomedica: OPG).

### 4.4. Statistical analysis

Consistent with the study hypothesis, all analyses were stratified according to PTH. We examined PTH as quartiles: ≤150, 150-300, 300-600, and >600 pg/mL. The characteristics of different PTH groups were compared using the chi-squared test for categorical variables and Student t test or ANOVA test for continuous variables, and using the Spearman correlation test analysis relation between serum albumin and bone turnover markers. The differences between groups were analyzed with the analysis of covariance (ANCOVA) test after adjusting for age and sex. The mean and standard deviation (SD) of each value were calculated for each group. The SPSS 18.0 statistical package was used for all statistical tests. Results with P < 0.05 were considered statistically significant.

## 5. Conclusions

Hypoalbuminemia, as a marker of chronic inflammation, differentially influences the bone markers OPG and P1NP in hemodialysis patients among different PTH groups. This might be related with inflammation related bone cells activation independent of PTH actions. Interestingly, this association was not seen in those with severely high PTH levels (PTH>600), which might be due to overwhelming effects of PTH and uremic toxins on bone cells. Future studies need to evaluate the validity of serum albumin in determining bone status among CKD and dialysis patients.

## Figures and Tables

**Table 1:** Demographic data of study patients according to PTH level (N=223).

Characteristic	PTH ≤150	PTH 150-300	PTH 300-600	PTH >600	p-value*	
	n=59	n=36	n=67	n=61		
Age (y)	69.9 ± 11.7	70.3 ± 11.2	66.6 ± 11.6	65.8 ± 13.9	0.1817	
Male	34 (57.6)	17 (47.2)	30 (44.7)	24 (39.3)	0.351	
Dialysis duration (months)	91.34±75.25	79.63±64.44	72.70±59.48	73.98±54.63	0.337	
BMI (kg/cm)	22.91±3.52	22.86±3.26	23.78±4.48	23.33±4.96	0.564	
Kt/V	1.48±0.27	1.51± 0.21	1.49± 0.22	1.49± 0.18	0.188	
nPCR (g/Kg/day)	1.27±0.31	1.30± 0.32	1.28±0.29	1.29± 0.33	0.254	
Laboratory measurements						
Albumin, g/L	3.6±0.4	3.7±0.6	3.4±0.4	3.3± 0.3^ab^	<0.0001	
Hematocrit, %	30.9±4.0	31.8±3.7	31.9±4.9	31.4 ± 4.7	0.6127	
Hemoglobin, g/dL	10.1±1.2	10.5±1.2	10.6±1.7	10.3 ± 1.6	0.3662	
Calcium, mg/dL	8.7±0.7	8.9±0.6	8.7±0.8	8.8 ± 0.9	0.6559	
Phosphate (P), mg/dL	4.1±1.3	4.9±1.1^a^	5.0±1.3^a^	5.1 ± 1.3^a^	0.0001	
IL6 (pg/ml)	4.02± 1.42	4.22± 1.48	4.52± 2.87	5.68± 3.21^abc^	<0.001	
FGF23 (pg/ml)	158.3±215.4	289.0±563.6	471.3±807.1^a^	395.3±756.1^a^	0.0158	
Bone Turnover Biomarkers						
PTH (pg/mL)	81.9±46.2	237.0±42.5^a^	412.7±83.2^ab^	1086.2 ± 425.1^abc^	<0.0001	
Alk-P (U/L)	97.5 ± 72.5	90.2 ± 42.8	93.3 ± 33.7	183.6 ± 242.9^abc^	0.0003	
OPG (pmol/L)	18.2±7.8	19.4±7.0	16.8±8.7	13.4±6.2^ab^	0.0005	
RANKL (pmol/L)	0.083±0.076	0.083±0.065	0.1±0.1	0.080±0.074	0.3109	
RANKL / OPG	0.007±0.001	0.005±0.004	0.008±0.011	0.009±0.010	0.427	
SOST (pg/mL)	161.0±276.4	114.2±131.7	118.6±115.0	97.8±99.6	0.7358	
DKK1 (pg/mL)	296.4±780.0	270.6±560.9	131.7±184.4	161.4±354.9	0.283	
Wnt10b (ng/mL)	3.0±1.4	3.4±1.4	3.9±1.2^a^	4.1±1.1^ab^	<0.0001	
Wnt16 (pg/mL)	30.2±29.5	29.9±38.9	56.9±68.0	43.3±58.6	0.0876	
P1NP (pg/mL)	127.9±131.2	110.3±150.3	153.494±154.886	186.0±179.0^b^	0.0138	
TRAP-5b (U/L)	4.9±3.3	4.3±2.6	4.5±2.1	8.2±6.1^bc^	0.0045	

*Categorical variables: chi-squared test; continuous variables: F test. ^a^ p<0.05 v.s iPTH<150; ^b^ p<0.05 v.s iPTH 150-300; ^c^ p<0.05 v.s iPTH 300-600. PTH, FGF23, RANKL, SOST, TRAP-5b, DKK1 have been log-transformed before analysis.

**Table 2:** Correlation between Serum Albumin and Bone Turnover Markers.

Variables	PTH≤150	PTH 150-300		PTH 300-600	PTH >600
IL-6	-0.231*	0.084		-0.217*	-0.316*
FGF23	-0.445^**^	-0.320		-0.421**	0.073
Phosphate (P)	0.125	0.022		-0.057	-0.142
Alk-P	-0.220	-0.358*		-0.143	-0.073
P1NP	-0.6^***^	-0.282		-0.241	0.013
TRAP5-b	-0.189	-0.367		-0.148	-0.311
OPG	0.344^**^	0.41^*^		0.257*	0.006
RANKL/OPG	-0.301^*^	-0.337		-0.205	0.18
Wnt10b	-0.261^*^	-0.321		-0.372**	0.128
SOST	-0.191	0.039		0.013	0.311*
DKK1	0.246	0.23		0.263*	-0.258

Spearman correlation test *p<0.05, **p<0.01, ***p<0.001.

**Table 3:** Bone Turnover Markers Changes in Hypo- and Normoalbuminemia HD Patients among Different Parathyroid Hormone Levels.

Variable	PTH≤150 (n=59)				p value^*^	PTH 150-300 (n=36)				p value^*^	PTH 300-600 (n=67)				p value^*^	PTH >600 (n=61)				p value^*^
	Albumin≤3.5 (n=27)		Albumin>3.5 (n=32)			Albumin≤3.5 (n=15)		Albumin>3.5 (n=21)			Albumin≤3.5 (n=40)		Albumin>3.5 (n=27)			Albumin≤3.5 (n=48)		Albumin>3.5 (n=13)		
	mean	±SD	mean	±SD		mean	±SD	mean	±SD		mean	±SD	mean	±SD		mean	±SD	mean	±SD	
Alk-P	97.7	±54.2	97.3	±85.8	0.980	109.4	±54.1	76.4	±26.2	0.042	94.2	±37.3	92.0	±28.1	0.794	198.2	±270.3	129.6	±69.8	0.371
FGF23	266.0	±271.9	67.4	±78.7	0.001	572.3	±781.6	86.6	±158.2	0.032	657.3	±906.4	202.8	±548.6	0.014	390.1	±839.8	414.3	±309.1	0.920
OPG	16.3	±9.1	19.8	±6.3	0.082	16.3	±3.8	21.6	±8.0	0.013	14.6	±7.8	20.0	±9.2	0.017	13.1	±6.1	14.6	±6.6	0.443
RANKL/OPG	0.008	±0.010	0.006	±0.008	0.277	0.005	±0.003	0.005	±0.005	0.977	0.009	±0.014	0.006	±0.006	0.236	0.009	±0.011	0.005	±0.006	0.229
SOST	115.1	±80.4	218.4	±404.7	0.330	102.9	±89.2	131.7	±185.0	0.620	133.2	±129.6	91.9	±77.7	0.210	98.5	±108.3	95.7	±67.5	0.940
DKK1	142.9	±210.8	241.82	±255.10	0.266	220.1	±554.9	303.4	±576.7	0.684	604.7	±2863.2	152.0	±127.2	0.364	187.2	±389.6	73.3	±178.9	0.354
Wnt10b	3.4	±1.3	2.7	±1.4	0.066	4.0	±0.9	2.9	±1.5	0.007	4.3	±0.8	3.2	±1.4	0.001	4.1	±1.1	4.4	±1.2	0.293
P1NP	185.5	±139.6	53.3	±69.4	0.001	162.3	±176.3	54.6	±93.3	0.05	183.5	164.7	96.3	±117.5	0.021	174.2	±178.5	229.6	±181.1	0.326
TRAP-5b	5.0	±3.7	4.7	±0.6	0.922	4.6	±2.6	4.3	±2.3	0.366	4.6	±1.7	4.3	±2.7	0.768	8.5	±6.3	6.5	±5.4	0.559

^*^ ANCOVA test 2.5. Changes in Bone Turnover Markers According to Serum Albumin levels in Different PTH Groups

## Abbreviations

BMI (kg/cm): body mass index (kilogram/centimeter)
Kt/V: dialyzer clearance of urea x dialysis time/volume of distribution of urea
nPCR(g/kg/day): normalized protein catabolic rate (gram/kilogram/day)
IL-6: interleukin-6
FGF-23: Fibroblast Growth Factor 23
PTH: parathyroid hormone
Alk-P: alkaline phosphatase-P
OPG: osteoprotegerin
RANKL: receptor activator of NF-kB ligand
SOST: sclerostin
DKK1: dickkopf-1
Wnt10b: wingless 10b
Wnt16: wingless 16
P1NP: procollagen type 1 N-terminal propeptide
TRAP-5b: tartrate-resistant acid phosphatase 5b
